# Efficient production of human acidic fibroblast growth factor in pea (*Pisum sativum *L.) plants by agroinfection of germinated seeds

**DOI:** 10.1186/1472-6750-11-45

**Published:** 2011-05-06

**Authors:** Yajun Fan, Wei Li, Junjie Wang, Jingying Liu, Meiying Yang, Duo Xu, Xiaojuan Zhu, Xingzhi Wang

**Affiliations:** 1 Institute of Genetics and Cytology, Northeast Normal University, Changchun 130024, China; 2 Department of Biology, Changchun Normal University, Changchun 130032, China; 3 Yunnan-Guizhou Plateau Institute of Biodiversity, Qujing Normal University, Qujing 655000, China; 4 College of Life Sciences, Jilin Agricultural University, Changchun 130118, China

## Abstract

**Background:**

For efficient and large scale production of recombinant proteins in plants transient expression by agroinfection has a number of advantages over stable transformation. Simple manipulation, rapid analysis and high expression efficiency are possible. In pea, Pisum sativum, a Virus Induced Gene Silencing System using the pea early browning virus has been converted into an efficient agroinfection system by converting the two RNA genomes of the virus into binary expression vectors for Agrobacterium transformation.

**Results:**

By vacuum infiltration (0.08 Mpa, 1 min) of germinating pea seeds with 2-3 cm roots with *Agrobacteria *carrying the binary vectors, expression of the gene for Green Fluorescent Protein as marker and the gene for the human acidic fibroblast growth factor (aFGF) was obtained in 80% of the infiltrated developing seedlings. Maximal production of the recombinant proteins was achieved 12-15 days after infiltration.

**Conclusions:**

Compared to the leaf injection method vacuum infiltration of germinated seeds is highly efficient allowing large scale production of plants transiently expressing recombinant proteins. The production cycle of plants for harvesting the recombinant protein was shortened from 30 days for leaf injection to 15 days by applying vacuum infiltration. The synthesized aFGF was purified by heparin-affinity chromatography and its mitogenic activity on NIH 3T3 cells confirmed to be similar to a commercial product.

## Background

The acidic mammalian fibroblast growth factor (aFGF) and the basic fibroblast growth factor (bFGF) bind to heparin decasaccharide and to domains of their tyrosine membrane spanning kinase receptor [1,2]. FGF is a powerful mitogen in many mammalian cell types. However its major importance is to switch endothelial cell growth to angiogenesis (formation of blood vessels) and to development of tumors [3,4].

Depending on the cell growth substrate FGF either stimulates endothelial cell growth or promotes capillary differentiation. Extensive cell spreading and growth were stimulated when the culture dishes were pre-coated with a high density of the extracellular matrix protein fibronectin (>500 ng/cm^2^) whereas lower coating densities (100-500 ng/cm^2^) resulted in cell shortening, cessation of growth and tube formation. Coating with different concentrations of type IV collagen or gelatin resulted in similar switches.

It is now recognized that oncogene induced excessive tumor cell proliferation is insufficient to produce a lethal tumor but requires simultaneous angiogenesis [3]. Tumor cell proliferation alone without angiogenesis frequently gives rise to dormant, microscopic tumors. The latter can be reactivated by increased angiogenic activity unless there is a permanent inhibition of this activity by endogenous angiogenesis inhibitors. Therefore there is interest in the efficient and cost effective production of recombinant FGFs for experiments in these areas.

In general, there are two major strategies for production of recombinant proteins of agricultural, nutritional or medical interest from genes introduced into plants: Stable transformation and transient expression. Transient expression has a number of advantages over stable transformation [5-13]. Simple manipulation, rapid analysis and high expression efficiency are possible. Primarily it avoids the extensive isolation, complex safety regulations and bureaucracy connected with growing stable transformed plants in the field. Previously tobacco, lettuce, tomato, cucumber and indica or japonica rice have been employed as transient expression hosts [14-20]. In addition some legume species such as alfalfa have been successfully used for production of monoclonal antibodies and blood substitutes [21]. Intact leaf vacuum system for transient expression of recombinant proteins by Agrobacterium was applied in 1997 to *Phaseolus acutifolius, Phaseolus vulgaris*, poplar and tobacco [16]. The vacuum was exerted on detached leaves and the plant materials were difficult to preserve fresh after inoculation. However, the transformation of other legume crops have been difficult and there is only one previous report of transient expression of recombinant proteins of medical or industrial interest in peas. Green et al. used this method to express three therapeutic proteins: hGH, HAWY1, and LicKM-PAD4 in Pisum sativum (green pea) varieties, the plants were grown for 7-14 days before vacuum infiltration [22]. We have used pea here and converted the pea early browning virus (PEBV) Virus Induced Gene Silencing System (VIGS) to an efficient agroinfection system [23]. PEBV belongs to the tobacco rattle virus genus, which has provided efficient silencing vectors for tobacco and tomato [24-28]. PEBV is a rod-shaped virus with a bipartite RNA genome [29]. The RNA1 molecule encodes all proteins required for replication and movement of the virus and can infect plants without RNA2. RNA2 encodes the viral coat protein and proteins needed for nematode transmission. PEBV has been modified into binary expression vectors for *Agrobacterium *transformation by inserting the expression cassettes of RNA1 and RNA2 between the right and left T-DNA borders of CAMBIA 1300 derived plasmids. Transcriptional control is exerted by a cauliflower mosaic virus 35S promoter and a nopaline syntase (NOS) terminator. Plasmid pCAPE1 contains the full-length cDNA of PEBV RNA1 with an intron inserted to stabilize the plasmid in bacteria. Plasmid pCAPE2-GFP contains the cDNA of PEBV RNA2 with the green fluorescent protein (GFP) coding sequence under the control of the coat protein promoter replacing the genes required for nematode transmission. Alternatively the genes for nematode transmission are replaced with the gene whose expression is desired, *in casu *the gene encoding human fibroblast growth factor. The two *Agrobacterium *strains with these two plasmids are mixed and the mixture delivered into the leaves with a syringe. (i) The plant RNA polymerase transcribes the full length cDNA of PEBV RNA1 including the encoded viral RNA polymerase from the T-DNA chromosomal insertions (ii) Transcription and translation of the RNA1 related genes permit the assembly of the viral components that spread the virus throughout the plant. (iii) The plant RNA polymerase transcribes simultaneously the pCAPE2 T-DNA chromosomal insertion encoding the virus coat protein and the targeted transgene. Translation provides the two proteins in the cells, i.e the desired recombinant protein and virus particles both spread throughout the plant.

Several different methods have been used to inoculate *Agrobacterium *into plants. Leaf injection is an effective way to introduce *Agrobacterium *containing genes of interest into plant leaves. We have previously used this method to express the human acidic fibroblast growth factor (aFGF) in tobacco leaves [15]. But leaf injection is laborious, and requires repeated injection of each plant leaf. Therefore leaf vacuum infiltration has been used for expression of β-glucuronidase in *Phaseolus vulgaris *[16] and for synthesis of tumor-specific single-chain and chimerical antibodies in tobacco leaves [30]. Vacuum infiltration has also been used to express an active human beta-interferon in lettuce and to transform lentil cotyledonary nodes and wheat inflorescence tissues [14,31,32].

We present here a useful transient plant expression system for recombinant proteins in pea plants by vacuum infiltration of germinating seeds with two Agrobacterium strains carrying the pCAPE1 and pCAPE2-GFP or pCAPE2-aFGF plasmid, respectively. Using GFP as reporter conditions were optimized and the procedure applied to synthesise human acidic fibroblast growth factor (aFGF) in pea plants. Western blot analyses confirmed the production of aFGF and a bioassay of mitogenic activity demonstrated that the purified recombinant aFGF was active in stimulating the growth of NIH 3T3 cells.

## Results

### Vacuum infiltration system for germinated pea seeds

**Figure 1:**
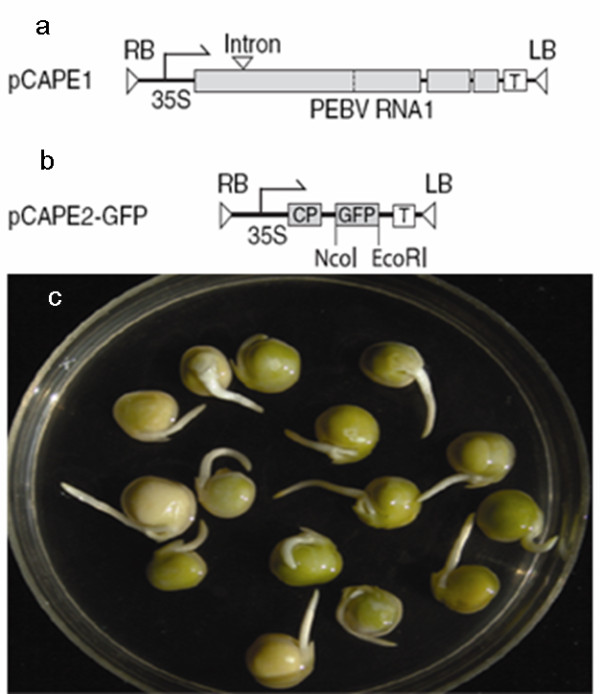
**Structure of the plasmids and the germinated pea seeds ready for agroinfection**. (a) pCAPE1 containing full-length cDNA of PEBV RNA-1 with an intron. (b) pCAPE2-GFP containing cDNA of PEBV RNA-2 with the GFP coding sequence replacing the genes required for nematode transmission. CP is the coat protein coding region. (c) The 36-48 hours germinated pea seeds with 2-3 cm roots were vacuum infiltrated with cultures of *Agrobacterium *containing binary plasmids pCAPE1and pCAPE2-GFP or pCAPE1 and pCAPE2-aFGF.

**Figure 2:**
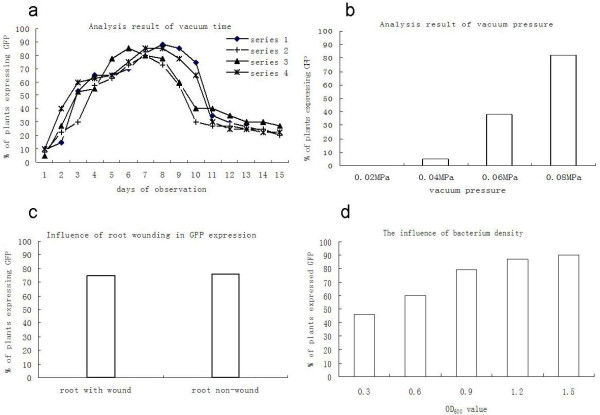
**Percentage of plants expressing GFP after vacuum infiltration of germinated pea seeds with a mixture of *Agrobacterium *GV3101 strains carrying binary plasmid pCAPE2-GFP and pCAPE-1 respectively**. (a) Frequency of transformants with variation of length of vacuum exposure. Series 1, 2, 3 and 4: peas seeds were subjected to vacuum for 1 min, 5 min, 10 min and 15 min. (b) The frequency of GFP expressing plants with increasing vacuum pressure (c) Frequency of GFP expressing plants with wounded and non-wounded roots. (d) The frequency of GFP expressing plants upon treatment with different bacterium suspension densities.

Pea seeds with 2-3 cm roots (Figure 1c) were exposed to a solution of ***Agrobacterium ***containing GFP encoding T-DNA at a density of OD_600 _= 1.0-1.5 and to a vacuum of 0.08 MPa for 1, 5, 10 or 15 min. At 8-10 dpi GFP fluorescence was observed. The spreading of fluorescence into newly formed leaves continued for 15 days when the plants were harvested and GFP expression was analyzed. The result indicated that the frequency of GFP expressing plants was independent of the times of vacuum treatment (Figure 2a). One min vacuum treatment was adopted for the following tests.

Vacuum pressure treatments were performed at 0.02, 0.04, 0.06 and 0.08 MPa using a cell density OD_600 _= 1.0-1.5, and a evacuation time of one min. The frequency of GFP expressing plants was 0, 5%, 38% and 82% at pressures of 0.02, 0.04, 0.06 and 0.08 MPa (Figure 2b) respectively, indicating that the vacuum pressure was a key factor for successful agroinfection. The highest number of transient expressing GFP plants were observed with the highest vacuum pressure tested (0.08 MPa). No GFP-positive plants were obtained at 0.02 MPa.

To determine if wounding affected the inoculation, germinated seeds with root wounding or with intact roots were immersed in *Agrobacterium *suspensions (bacterium density OD_600 _= 1.0-1.5) and vacuum applied for 1 min at 0.08 MPa vacuum pressure. The frequency of GFP expressing was 74.6% and 75.7% in plants inoculated with and without root wounding indicating that wounding did not affect the induction of GFP expression (Figure 2c).

Our data indicated that bacterium suspension density was another important factor influencing the frequency of induction of GFP expression. Bacterium suspension densities of 0.3, 0.6, 0.9, 1.2 and 1.5 (OD_600_) were used for inoculation at 0.08 MPa vacuum pressure for 1 min. The percentage of GFP expressing plants was above 79% with a suspension density exceeding 0.9 OD_600 _(Figure 2d).

### Comparison of GFP expressing plants obtained with vacuum infiltration of germinated seeds with Agrobacterium versus leaf injected plants

**Figure 3:**
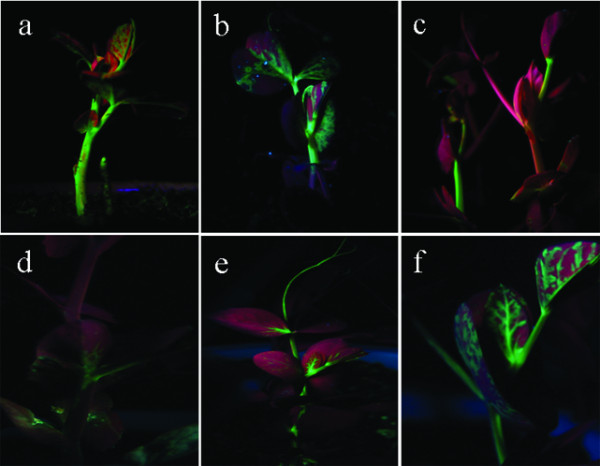
**Examples of GFP expression in pea plants after agroinfection with the two agrobacterium strains GV3101 one carrying pCAPE2-GFP and the other pCAPE1**. a, b and c: agroinfection plants produced by vacuum infiltration of germinated seeds, d, e and f: agroinfection plants obtained by leaf injection. (a) 9 dpi, (b) 12 dpi, (c) 26 dpi, (d) 9 dpi, (e) 11 dpi, (f) 12 dpi.

Germinated pea seeds were vacuum infiltrated (0.08 MPa, 1 min, OD_600 _= 1.2) or injected into the backside of the young leaves on 2-week-old pea plants using a 1-ml syringe (OD_600 _= 1.2). GFP fluorescence was monitored at 8-10 dpi. In the vacuum infiltrated seedlings GFP was first expressed in the stem and reached its highest value in both leaves and stems at 12-15 dpi (Figure 3a-3c). With leaf injection, GFP fluorescence was first visualized in treated leaves at 8-10 dpi (the same as with vacuum infiltration of germinated seeds), and GFP was detected in stems and upper non-agroinfected leaves on the next day. In the following two days the diffused GFP was more extensive in leaves (Figure 3d-3f). The amount of GFP tended to decrease with both of the treatments at 15-16 dpi. At 18-20 dpi the GFP fluorescence had almost disappeared. However, there was another peak of GFP expression in the upper stems in vacuum infiltrated plant leaves 6-7 days later.

### GFP and aFGF protein analysis

**Figure 4:**
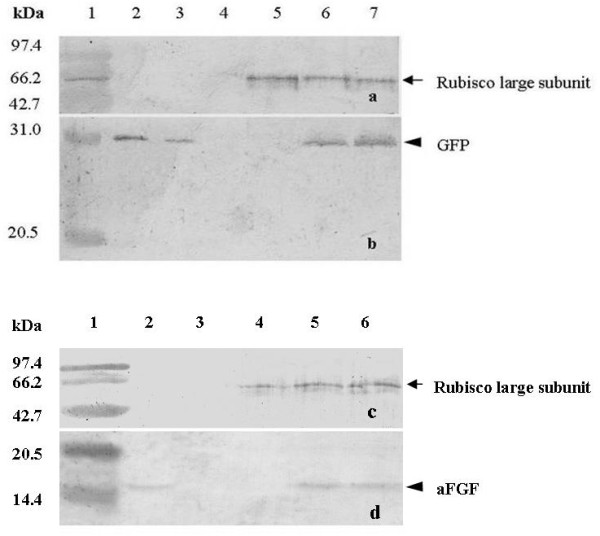
**Western blot of the total soluble protein extracts from plants agroinfected with both GV3101 carrying pCAE1 and GV3101 carrying pCAE2-GFP**. (a and c) Western blot probed with antibody against RUBISCO large subunit as control for the amount of proteins loaded. (b) Proteins probed with anti-GFP polyclonal antibody. Both for a and b: Lane 1, low-molecular marker; lanes 2 and 3, purified GFP proteins, lane 4, proteins from bacteria; lane 5, proteins from non-inoculated pea plants; lane 6, proteins from plants agroinfected by leaf injection, lane 7, proteins from plants agroinfected by vacuum infiltration. (d) Western blot of the total soluble protein extracts from the plants agroinfected with GV3101-pCAE1 and GV3101-aFGF probed with anti-aFGF antibody. Both for c and d: lane 1, low-molecular marker; lane 2, commercial aFGF; lane 3, proteins from bacteria; lane 4, proteins from non-inoculated pea plants; lane 5, proteins from plants agroinfected by leaf injection; lane 6, proteins from plants agroinfected by vacuum infiltration.

Western blot analysis was used to compare the two methods. The total soluble proteins in 20 plants agroinfected with GV3101-pCAE2-GFP and GV3101-pCAE1 were extracted. The results showed single bands for GFP or aFGF by Western blot (Figure 4a-4d). The amount of GFP or aFGF expressed in vacuum-infiltrated plants was similar to that obtained by leaf injection: GFP was 1% of the plant total soluble protein.

### Mitogenic activity of pea plant-derived aFGF protein

**Figure 5:**
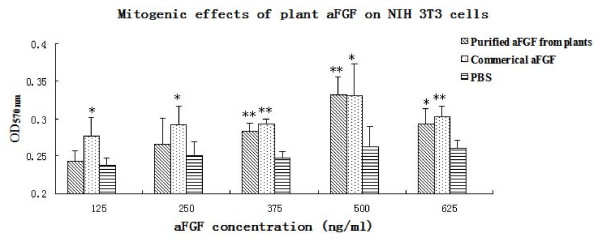
**Induction of mitogenic activity of NIH 3T3 cells by pea plant-derived aFGF**. Commercial or plant expressed aFGF in PBS (20 mmol/l sodium phosphate (pH 7.4) containing 0.6 mol/l NaCl) was used as postive control and PBS as negative control. *p < 0.05,** *p *< 0.01 as compared with PBS control.

The activity of the pea plant-derived acid fibroblast growth factor (aFGF) was determined by a bioassay determining the mitogenic activity elicited in NIH 3T3 cells. Concentrations of aFGF in the NIH 3T3 cell cultures ranged from 125 to 625 ng/ml. The growth of 3T3 cells were stimulated significantly both by pea plant-derived aFGF and the commercial aFGF (Figure 5). The highest stimulation was obtained at 50 ng aFGF added to 100 μl cell culture.

## Discussion

### Importance of endothelic growth factors

With the discovery that oncogene induced excessive tumor cell proliferation is angiogenesis dependent the angiogenesis inducing activity of acid and basic FGFs as well as that of the vascular endothelic growth factor (VEGF) has become of great interest. Efficient and cost-effective production of these growth factors and other proteins involved in neovascularization are of prime importance in the analysis of the function of natural and synthetic inhibitors of the network of capillary tubes stimulated by these growth factors [3]. Tumor cell proliferation in the absence of angiogenesis can give rise to dormant microscopic tumors of 1 mm^3 ^or less but these in situ cancers are harmless to the host. However a switch to the angiogenic phenotype permits these dormant cancers to become rapidly growing tumors that can subsequently metastasize [4,33,34].

The gene participants in the angiogenic switch have recently been determined [33]. Human dermal endothelial cells isolated from two different human donors were treated for 4 h with (i) Vascular endothelial growth factor (VEGF) (10ng·ml^-1^), (ii) bFGF (20ng·ml^-1^) (iii) combined VEGF plus bFGF and (iv) endostatin (200 ng·ml^-1^). After isolation of total RNA from these cells upregulated and downregulated transcripts by the treatments were determined by microarray analysis of 74,834 human cDNA clones. Expression levels of the RNA transcripts were quantitated by real time PCR. After treatment with endostatin 1,230 genes were significantly upregulated. These same genes were simultaneously down regulated after treatment with bFGF, and VEGF+BFGF and are thus considered participants in anti-angiogenic signaling. Conversely 1,140 transcripts downregulated by endostatin were found upregulated after VEGF, bFGF and VEGF+bFGF treatment and are therefore considered participants in pro-angiogenic signaling.

FGFs including aFGF and bFGF are implicated in maintenance of tumors often by mediating angiogenesis [35]. aFGF is a growth factor for capillary blood vesselsand involved in the stimulation of DNA synthesis and the proliferation of a wide variety of cell types [36]. It plays an important role in morphogenesis, angiogenesis and wound healing [2,15,37].

### A method for expressing large amounts of recombinant proteins in higher plants

In 1997 an intact leaf vacuum infiltration system for transient expression of recombinant proteins by Agrobacterium was applied to *Phaseolus acutifolius, Phaseolus vulgaris*, poplar and tobacco [16]. Vacuum was exerted on detached leaves and the plant materials were difficult to preserve fresh after inoculation. Green et al. used this method to express three therapeutic proteins: hGH, HAWY1, and LicKM-PAD4 in Pisum sativum (green pea) varieties. In contrast to our method the plants were grown for 7-14 days before vacuum infiltration [22]. In our study vacuum infiltration was applied to germinated pea seeds with 2-3 cm roots and factors influencing expression efficiency such as vacuum pressure, vacuum duration and bacterial density have been optimized. The optimal conditions for transient expression were a vacuum pressure at 0.08 MPa for 1 min with a bacterial OD_600 _greater than 0.9. These parameters were decisive factors causing 80% of the treated plants to produce the desired recombinant GFP.

## Conclusions

When the modified pea early browning virus genomes with the targeted marker GFP were multiplied and spread in pea plants after inoculation, the GFP was concomitantly expressed at a high level. In the vacuum infiltrated seedlings GFP was first expressed in the stem and thereafter in leaves and reached its highest value in both leaves and stems at 12-15 dpi. The vacuum infiltration method, gives similar high yields to leaf injection but is more efficient. Time consuming leaf injection was avoided and the production cycle of plants was shortened from 30 days to 15 days. Mitogenic activity analysis demonstrated that the purified aFGF from pea plants stimulated the growth of NIH 3T3 cells. It is concluded that the reported procedure can provide large-amounts of functional recombinant proteins in pea and possibly other plants of interest. It can also be employed for virus induced gene silencing to study gene functions in a more effective way.

## Methods

### Plasmid construction

The unsuitable *Nco*I site of the pCAPE2 vector was modified to synthesise a *Bgl*II for insertion of the *aFGF *(A01474) sequence from the pET28-haFGF construct (provided by Dr X. Li, Jilin Agricultural University, Changchun, China). Forward (F) and reverse (R) primers to amplify the *aFGF *fragment with unique *Bgl*II and *Eco*RI sites were designed: 5'-GGAAGATCTACATGGCTGAAGGGGAAATCAC-3'(F) and 5'-CGGAATTCTTAATCAGAAGAGACTGGCAGG-3'(R). The *Bgl*II-*Eco*RI PCR fragment was subcloned into pCAPE2 by replacement of the GFP sequence (Figure 1a, 1b show the PEBV vectors), and the new vector was named pCAPE2-aFGF.

### Plant materials and growth condition

American dwarf pea *P. sativum *was obtained from the local market. The seeds were soaked in water for 6-8 hours and germinated on moist filter paper in the dark for 36-48 hours at 25°C. A part of germinated seeds were then vacuum infiltrated (see details below), and the others were planted in pots for leaf injection. Plants were grown in a plant growth chamber at 25°C under a 16 h cool fluorescent light/8 h dark cycle.

### Plasmid introduction to Agrobacterium

The binary vectors pCAPE2-aFGF, pCAPE2-GFP and pCAPE1 were introduced into *Agrobacterium tumefaciens *strain GV3101 by the freeze-thaw method [38]. Individual clones were grown in 200 ml LB with 50 μg· ml^-1 ^rifampicin, 50 μg·ml^-1 ^kanamycin and 25 μg·ml^-1 ^tetracycline at 28°C for 12-16 h with shaking. At OD_600 _= 1.0-1.5 the bacteria were harvested by centrifugation (3500 g) at room temperature. Cells were resuspended in infiltration medium (10 mM NaCl, 1.75 mM CaCl_2_, 100 μM acetosyringone and 250 μg·L^-1 ^Tween 20, pH 5.6) and incubated at room temperature for 90 min without shaking.

### Agrobacterium vacuum infiltration procedure

*Agrobacterium *culture harboring pCAPE1 was mixed with culture harboring pCAPE2-GFP 1:1 v/v and infiltration medium. Alternatively *Agrobacterium *culture with pCAPE1 was combined with culture containing pCAPE2-aFGF 1:1 v/v with infiltration medium, prior to infiltration. Vacuum pressure, vacuum period, root wounding and bacterium suspension density were selected to optimize the induction of transient expressing plants by using the vacuum desiccator SHZ-D Ⅲ (Yuhua Inc., Henan, China). Triplicate experiments with 20 seeds in each were carried out. Germinated seeds with roots of 2-3 cm were submerged in *Agrobacterium *suspensions (Figure 1). When the wounding treatment was used, the roots was pricked with a needle 1 to 2 times, whereafter the continuous vacuum was applied. The germinated seeds were planted in pots and 8 to 10 days post inoculation, GFP fluorescence was observed in seedlings using a 100 W, long-wave UV lamp (Black Ray model B-100 AP; Ultra-Violet Products, Upland, CA, U.S.A.). Plants were photographed with a digital camera (Sony DSC-F828) mounted with yellow filters. In all the optimization treatments the percent of plants expressing GFP (GFP expression frequency) was used to estimate the treatment efficiency during 20 days post expression period. Acidic fibroblast growth factor (aFGF) was expressed in pea plants using the optimized seed vacuum infiltration system.

### Comparison of the expression level in vacuum infiltrated germinated seeds versus leaf injection methods

Leaf injection was performed on 2-3 week old pea plants using a 1-ml syringe without needle (OD_600 _= 1.2), The pea plants treated were then placed in the dark in a humid atmosphere for 24 h to recover from the treatment, whereas vacuum infiltration was conducted on the germinated seeds with 2-3 cm roots. Plants were monitored at 8-10 dpi and photographed. A total of 20 plants were harvested in each treatment and the total soluble proteins were extracted from pea plant leaves and stems. Western blot was used for determining the expression level and Bandscan software (Glyko Inc., USA) was used for estimating the amount of GFP.

### Western blot analysis of GFP and aFGF

The plant material (8-10 dpi) were frozen in liquid nitrogen and pulverized. The powder was stirred in extraction buffer of 20 mM sodium phosphate (pH 7.4) containing 0.6 M NaCl, then centrifuged at 20817 g for 20 min at 4°C. The supernatants were analyzed by 15% SDS-PAGE and then transferred to nitrocellulose membranes for immunoblotting. The murine polyclonal antibodies against GFP, Rubisco large subunit and aFGF were made in our laboratory. Antibodies bound to GFP protein, Rubisco large subunit protein and aFGF protein were visualized by chemiluminescent detection (SuperSignal WestPico Trial kit from Pierce). The proteins were extracted from GV3101-inoculated and control pea plants. The Western blot image was used to analyze protein expression quantity using Bandscan software.

### Purification and mitogenic activity analysis of pea plant-derived aFGF

The pea plant-derived aFGF was purified by heparin-affinity chromatography. A heparin-Sepharose Column was washed with equilibration buffer (0.6 M NaCl containing 20 mM sodium phosphate (pH7.4)) and the total soluble protein of the Agroinfected plants applied. Bound proteins were eluted with elution buffer (1.2 M NaCl containing 20 mM sodium phosphate (pH7.4)). The purified protein was then bioassayed for mitogenic activity. Commercial aFGF was used as the positive control and equilibration buffer was used as the negative control. NIH 3T3 cells (purchased from Shanghai Institute of Cell Biology, Chinese Academy of Sciences, Shanghai) were grown in IMDM (Gibco BRL, USA) medium supplemented with 10% (v/v) fetal bovine serum (Gibco BRL, USA). When the culture reached the mid-exponential phase, cells were transferred to a 96-well plate (5000 cells/well) and incubated in IMDM medium for 24 h and 12.5, 25, 37.5, 50, and 62.5 ng aFGF were added to 100 μl cell culture respectively. Four repeats of each treatment were made. After 24 h incubation 20 μl of MTT [3-(4, 5-dimethylthiazol-2-yl)-2, 5-diphenyl-2*H*-tetrazolium bromide; 5 mg·ml^-1^] were added to each well and incubation continued for 6 h to determine the number of viable cells. After discarding the medium, 150 μl of DMSO was added to each well, and the plate was kept at 37°C for 30 min. The viable cell number was measured by the absorption of 570 nm as described in [15].

## Authors' contributions

YF designed the study, performed the statistical analysis and drafted the manuscript. WL carried out the immunoassays. JW and JL purified aFGF protein and analyzed mitogenic activity. MY and DX constructed the plasmid, and dealt with the plant materials. XZ and XW conceived of the study, and participated in its design and coordination and helped to draft the manuscript.
